# Functional and proteomic analysis of *Lactobacillus rhamnosus*-derived extracellular vesicles with antioxidant and anti-inflammatory activity

**DOI:** 10.1038/s41598-025-32989-6

**Published:** 2025-12-18

**Authors:** Hyesoo Wang, Junbo Sim, A. Hyung Jeong, Hayeon Kim, Yeji Im, Kyung-Baeg Roh, Eunsun Jung, Deokhoon Park, Eunae Cho

**Affiliations:** BioSpectrum Life Science Institute, A1805, U-TOWER, 767, Sinsu-ro, Suji-gu, Yongin-si, 16827 Gyeonggi-do South Korea

**Keywords:** Extracellular vesicles, Lactic acid bacteria, Cell-derived vesicles, Anti-oxidant, Anti-inflammation, Biochemistry, Biological techniques, Biotechnology, Microbiology, Molecular biology

## Abstract

Extracellular vesicles (EVs) derived from lactic acid bacteria have gained attention as mediators of host-microbe interaction. However, their molecular characteristics compared to cell-derived vesicles (CDVs) remain poorly understood. In this study, we isolated EVs and CDVs from *Lactobacillus rhamnosus* and characterized them throughout proteomic and functional analyses. Both vesicle types displayed spherical morphology as observed by nanoparticle tracking analysis and transmission electron microscopy. Western blotting revealed distinct protein profiles, with HSP70 specifically detected in EVs. Proteomic profiling using LC-MS/MS identified a total of 1,342 proteins, including 114 unique to CDVs. Differential expression proteins analysis showed 70 proteins upregulated and 81 downregulated in EVs. Gene Ontology (GO) analysis indicated that EVs were enriched in proteins related to signal transduction, environmental adaptation, and transmembrane transport, while CDVs showed enrichment in ribosomal and nucleic acid metabolism-related proteins. Functional assays further demonstrated that EVs exhibited significantly stronger antioxidant and anti-inflammatory activities than CDVs. These findings suggest that *L. rhamnosus*-derived EVs possess distinct proteomic signatures and superior bioactivity, supporting their potential role in probiotic-based therapeutic strategies, particularly for skin health.

**Supplementary Information:**

The online version contains supplementary material available at 10.1038/s41598-025-32989-6.

## Introduction

The gastrointestinal tract, skin, and oral cavity are inhabited by diverse microbial communities that play critical roles in maintaining host health. Among these, gut microbiota has been extensively studied for its roles in immune modulation, metabolism, and disease prevention. The Food and Agriculture Organization and World Health Organization define probiotics as “live microorganisms that confer health benefits to the host when administered in adequate amounts.” Lactic acid bacteria (LAB), particularly the genus *Lactobacillus*, are widely recognized probiotics. *Lactobacillus rhamnosus* (LR), a Gram-positive, non-spore-forming bacterium, is notable for its tolerance to acid and bile, allowing it to survive and adhere in the gastrointestinal tract. The LR strain GG (LGG) has been widely studied for its effects in alleviating symptoms of irritable bowel syndrome, modulating immune responses, and supporting intestinal health.

Extracellular vesicles (EVs) are membrane-bound nanoparticles (50–200 nm in diameter) released by various cell types and are involved in intercellular communication through the delivery of proteins, lipids, and nucleic acids. While most EVs research has focused on mammalian cells, EVs are also secreted by bacteria and plant cells^1,2^. EVs from bacteria, such as those from *E. coli*, have been studied since the 1960s^3,4^ and are now known to carry bioactive molecules^5^. EVs derived from LAB have recently gained attention for their roles in host-microbe interactions, exhibiting antimicrobial, immunomodulatory, and anti-inflammatory effects^6,7^. For example, *L. acidophilus*-derived EVs deliver bacteriocins to suppress pathogens^8^ and *L. paracasei* EVs transport functional proteins such as P40 and P75, which contribute to promote tissue repair^9^. EVs from *L. plantarum* have also shown protective effects in atopic dermatitis models^10^. Despite their therapeutic potential, challenges such as large-scale production, safety assessment, and mechanistic understanding remain to be addressed for their clinical application.

**Fig. 1:**
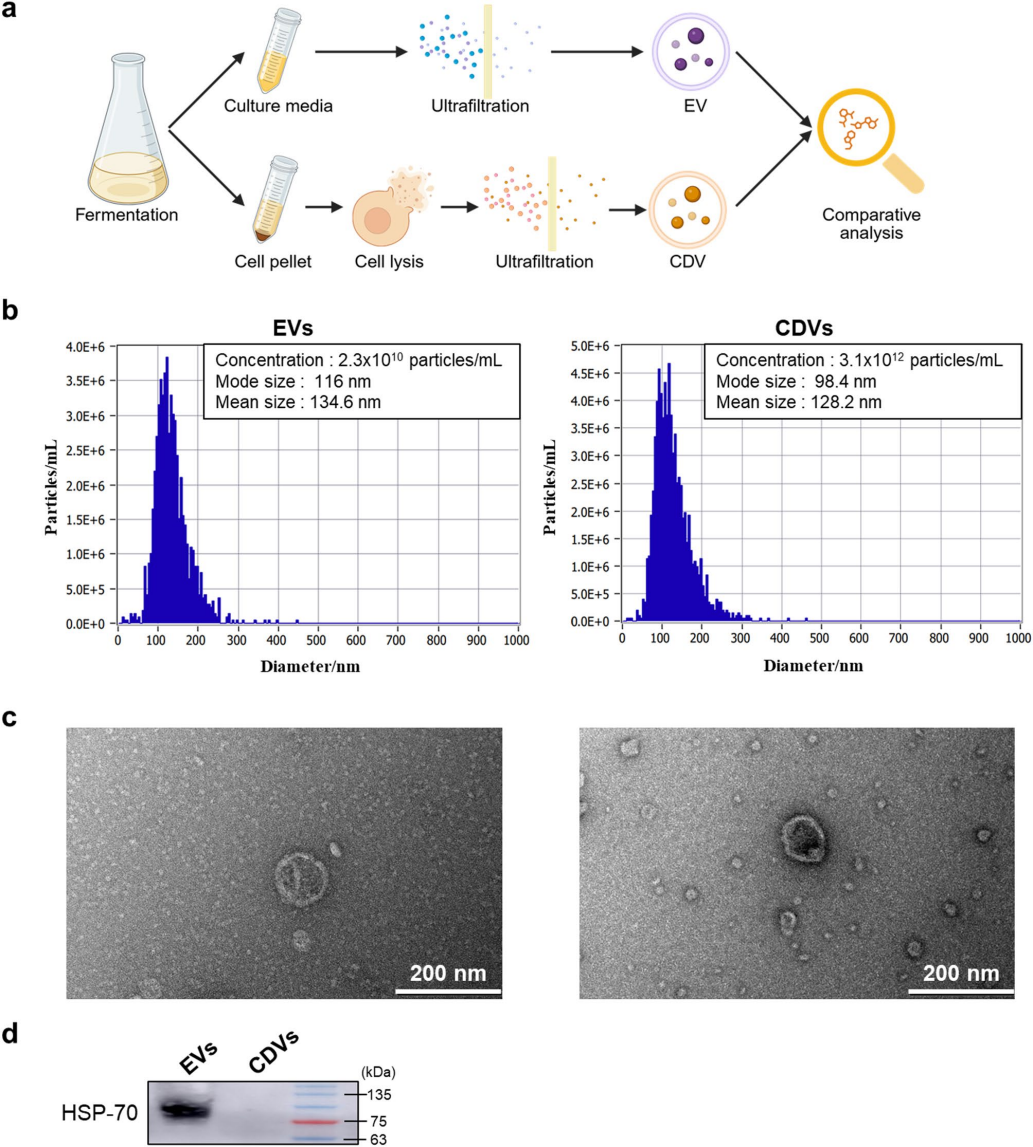
Characterization of EVs and CDVs. (**a**) Schematic illustration of the experimental workflow. (**b**) Size distribution and particle concentration of EVs (left) and CDVs (right) derived from LR measured using the ZetaView system. The X-axis represents particle size (nm) and the Y-axis indicates particle concentration. (**c**) TEM images of purified EVs (left) and CDVs (right). Scale bars, 200 nm. (**d**) Western blot analysis of HSP70 in EVs and CDVs. Molecular weight marker shown on the right.

More recently, cell-derived vesicles (CDVs), which are generated through physical disruption or extrusion of cells, has gained increasing attention. According to the 2023 MISEV(Minimal information for studies of extracellular vesicles) guidelines, these EVs-mimetic vesicles are classified as artificial CDVs^11^. While EVs are naturally secreted into extracellular environments, CDVs remain within cells unless actively extracted. CDVs have been reported to offer higher production yields and exhibit therapeutic efficacy comparable to EVs^12,13^. However, to date, no direct comparison has been made between LR-derived CDVs and EVs. In this study, we performed a comparative analysis of EVs and CDVs isolated from LR, a probiotic strain commonly used in skin and health applications. The overall experimental workflow is shown in Fig. 1a. Proteomic profiling and functional assays were employed to evaluate their molecular cargo, biological activity and delivery efficiency. These findings provide insights into the potential of LAB-derived nanovesicles as next-generation therapeutic agents.

## Results

### Characterization of EVs and CDVs

Nanoparticle Tracking Analysis (NTA) was conducted using ZetaView software to determine the size and concentration of EVs and CDVs. Both vesicles types ranged from 50 to 200 nm in diameter. Ther mode size was 116 nm for EVs and 98.4 nm for CDVs. The particle concentration was 2.30 × 10^10^ particles/mL for EVs and 3.10 × 10^12^ particles/mL for CDVs (Fig. 1b). Transmission electron microscopy (TEM) analysis revealed that both EVs and CDVs exhibited spherical, cup-shaped morphology and were enclosed by a lipid bilayer (Fig. 1c).

Western blotting was performed to analyze the protein markers of the vesicles. HSP70, an established EV marker, was detected at 70 kDa exclusively in EVs, confirming the identity of extracellular vesicles (Fig. 1d). The original blot is presented in Supplemental Figure S1.

### Proteomic profiling of EVs and CDVs

#### Venn diagram analysis

**Fig. 2:**
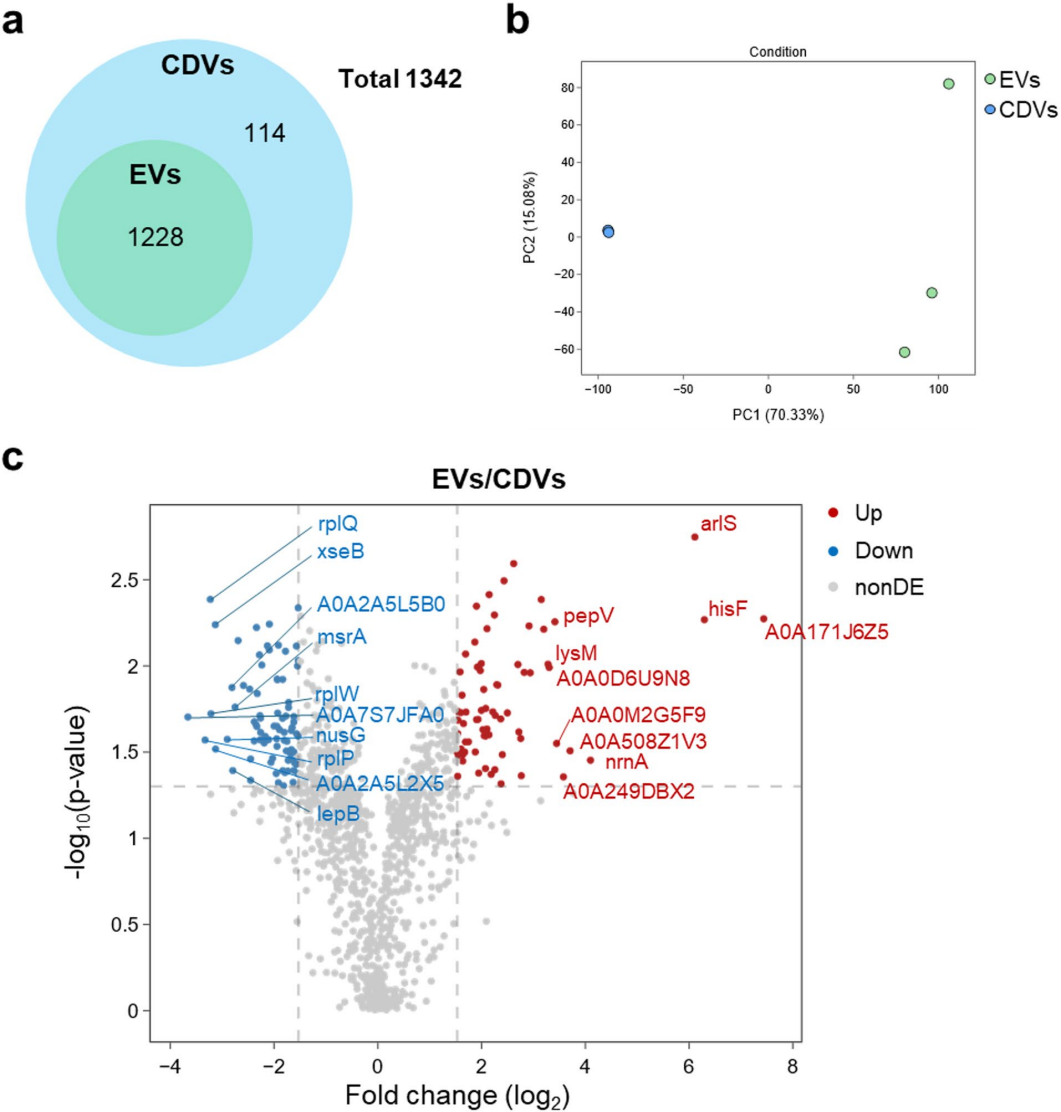
Comprehensive proteomic analysis of EVs and CDVs. (**a**) Venn diagram illustrating the overlap and unique protein distributions between EVs and CDVs, with 1,228 shared proteins and 114 unique to CDVs out of 1,342 identified. (**b**) PCA showing distinct clustering between EVs and CDVs; PC1 accounts for 70.33% of total variance. (**c**) Volcano plot of DEPs between EVs and CDVs (|log₂FC| > 1.54, *p* < 0.05). Red and blue dots represent proteins significantly upregulated (*n* = 70) and downregulated (*n* = 81) in EVs, respectively.

A total of 1,342 proteins were identified from both EVs and CDVs samples. Among them, 1,228 proteins (91.5%) were shared between the two vesicles types, and 114 proteins (8.5%) were unique to CDVs, with no proteins found exclusively in EVs. The overall distribution of shared and unique proteins is illustrated in the Venn diagram (Fig. 2a).

#### Principal component analysis (PCA)

PCA demonstrated clear separation between EVs and CDVs groups. The first principal component (PC1) accounted for 70.33% of the variance and distinguished the two groups, whereas PC2 explained an additional 15.08% and accounted for intra-group variability (Fig. 2b).

#### Identification of differentially expressed proteins (DEPs)

To further investigate proteomic differences, DEPs were identified based on the criteria of |log₂ fold change| > 1.54 and *p* < 0.05. A total of 151 proteins were differentially expressed between EVs and CDVs. Among these, 70 proteins were significantly upregulated and 81 were downregulated in EVs relative to CDVs (Fig. 2c).

#### Gene ontology (GO) enrichment analysis

**Fig. 3:**
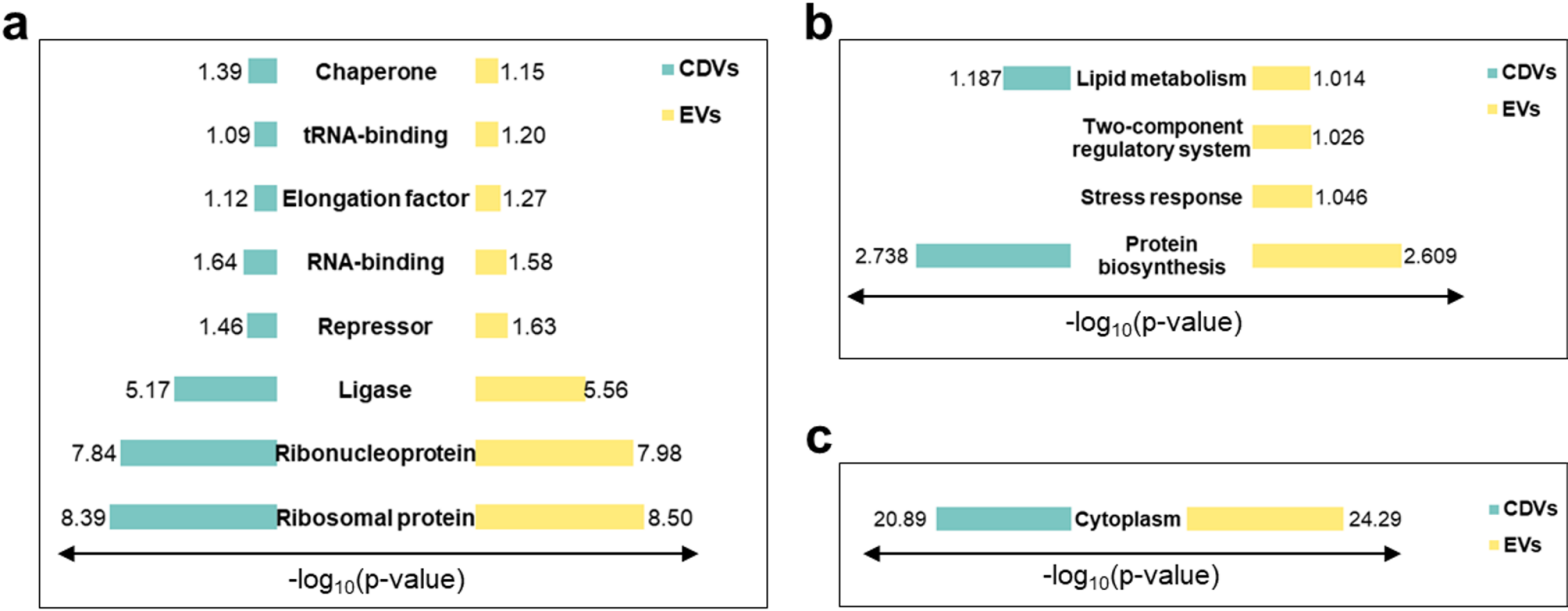
Gene Ontology (GO) enrichment analysis of EVs and CDVs. Top enriched GO terms (threshold: *p* < 0.1) are shown for (**a**) molecular function, (**b**) biological process, and (**c**) Cellular component categories.

GO enrichment analysis revealed distinct molecular features between EVs and CDVs. In the molecular function category, both vesicle types showed comparable enrichment patterns. EVs consistently exhibited slightly higher -log₁₀(p) values across most terms, indicating marginally stronger enrichment compared to CDVs (Fig. 3a). In the biological process category, proteins related to the two-component regulatory system and stress response were detected only in EVs (Fig. 3b). In terms of cellular components, cytoplasmic proteins were significantly enriched in both EVs and CDVs, with EVs showing higher enrichment (Fig. 3c).

#### Expression patterns of upregulated and down regulated proteins in EVs

Table 1 summarizes the top ten most significantly upregulated and downregulated proteins in EVs compared to CDVs, based on log₂ fold change and *p*-value. Among the upregulated proteins, several were associated with signal transduction and environmental adaptation including *hisF* (L-histidine biosynthesis kinase), *arlS* (a major transducer of extracellular signals), and *nrnA* (involved in microbial metabolism in diverse environments). In contrast, the most downregulated proteins in EVs comprised multiple ribosomal proteins (e.g., *rplP*, *rplQ*, and *rplW*) and transcriptional regulators (e.g., A0A2A5L5B0). Supplemental Table S1 presents the median expression levels (log2) of each protein in EVs and CDVs, allowing readers to evaluate the individual abundance of proteins in each vesicle type.

**Table 1:** Mostly highly common differentially up-regulated and down-regulated proteins in EVs.

No.	Protein ID	Gene	Description	Log2FC	*P* value
**Up-regulated proteins**					
1	A0A171J904	hisF	Kinase, L-histidine biosynthetic process	6.294786	0.005393
2	A0A6N3BJ42	arlS	Kinase, Transferase, key signal transducers	6.111297	0.001787
3	A0A508YJH6	nrnA	Nucleic acid binding, Microbial metabolism in diverse environments	4.100688	0.035235
4	A0A508Z1V3		NADP-dependent oxidoreductase	3.706757	0.031091
5	A0A249DBX2		MFS Transporter, Transmembrane transporter activity	3.578419	0.044009
6	A0A0M2G5F9		DUF2140 domain-containing protein	3.446402	0.028171
7	A0A508Z0V2	pepV	Dipeptidase activity	3.414005	0.00555
8	A0A0D6U9N8		Sugar transport	3.303865	0.010212
9	A0A7 × 2J725	lysM	Binds to peptidoglycan, Cell wall binding	3.281652	0.009773
10	A0A508YSK0	menA	1,4-dihydroxy-2-naphthoate polyprenyltransferase	3.199538	0.006125
**Down-regulated proteins**					
1	A0A7S7JFA0		Membrane protein	-3.65959	0.019784
2	A0A0D6U8P4	rplP	rRNA binding, tRNA binding, Translation	-3.32925	0.026909
3	A0A0D6U9Y8	rplQ	Structural constituent of ribosome, Tranlation	-3.22756	0.004112
4	A0A809N128	rplW	rRNA binding, Structural constituent of ribosome, Translation	-3.21578	0.018939
5	A0A2A5L762	xseB	Exodeoxyribonuclease	-3.13193	0.005765
6	A0A2A5L2 × 5		RNA binding	-3.1263	0.030361
7	A0A0D6U5L6	nusG	Transcription	-2.90121	0.026647
8	A0A2A5L5B0		DNA binding, Transcription regulator	-2.81033	0.013346
9	A0A180C927	lepB	Signal peptide processing	-2.79495	0.040458
10	A0A171J940	msrA	Protein modification process, Oxidoreductase	-2.75188	0.017343

### Antioxidant effects of EVs and CDVs

The antioxidant activities of EVs and CDVs were evaluated using the DPPH radical scavenging, cell viability under oxidative stress, and intracellular reactive oxygen species (ROS) assays.

**Fig. 4:**
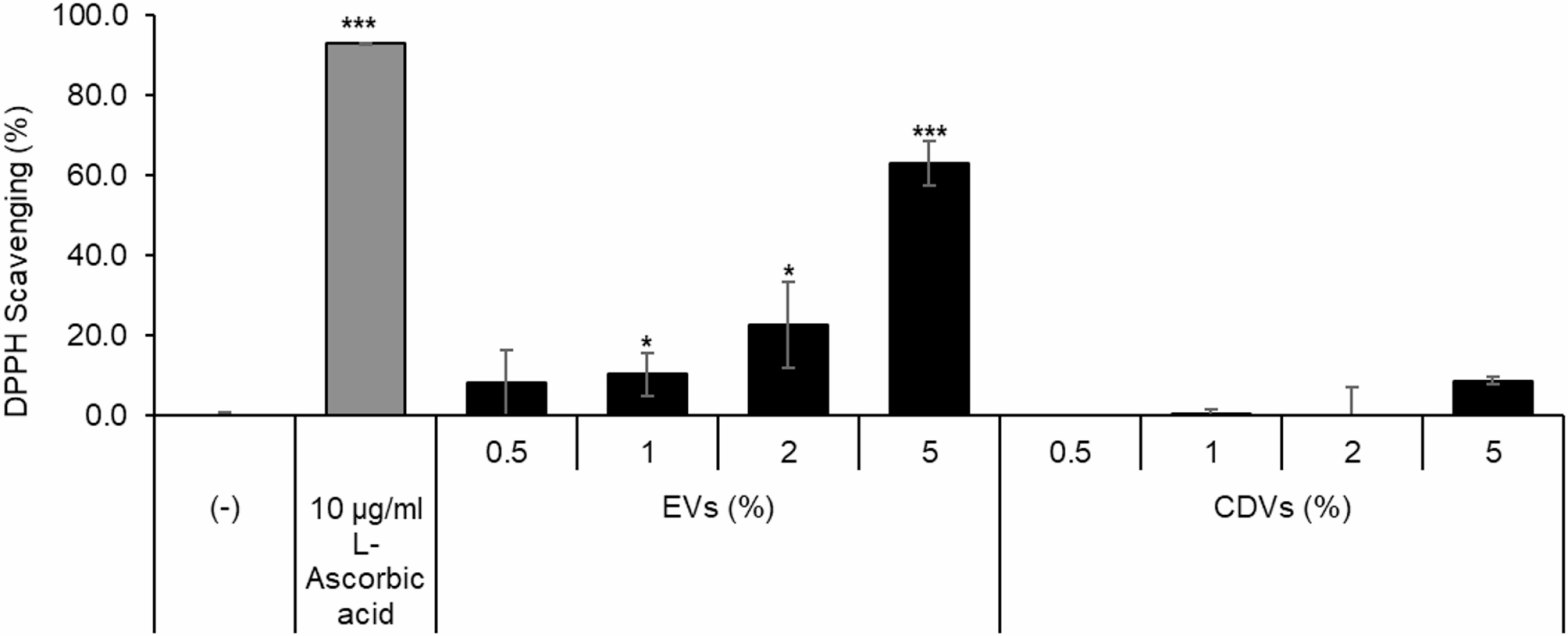
Antioxidant activity of EVs and CDVs measured by DPPH assay. L-ascorbic acid (10 µg/mL) was used as a positive control. Data are presented as mean ± SD of three independent experiments. *p < 0.05, **p < 0.01, ***p < 0.001 vs. control.

In the DPPH assay, EVs exhibited significantly higher radical scavenging activity than CDVs. At a concentration of 5%, EVs showed 62.98 ± 5.56% scavenging activity, whereas CDVs showed 8.67 ± 0.95%. Ascorbic acid was used as a positive control (Fig. 4).

**Fig. 5:**
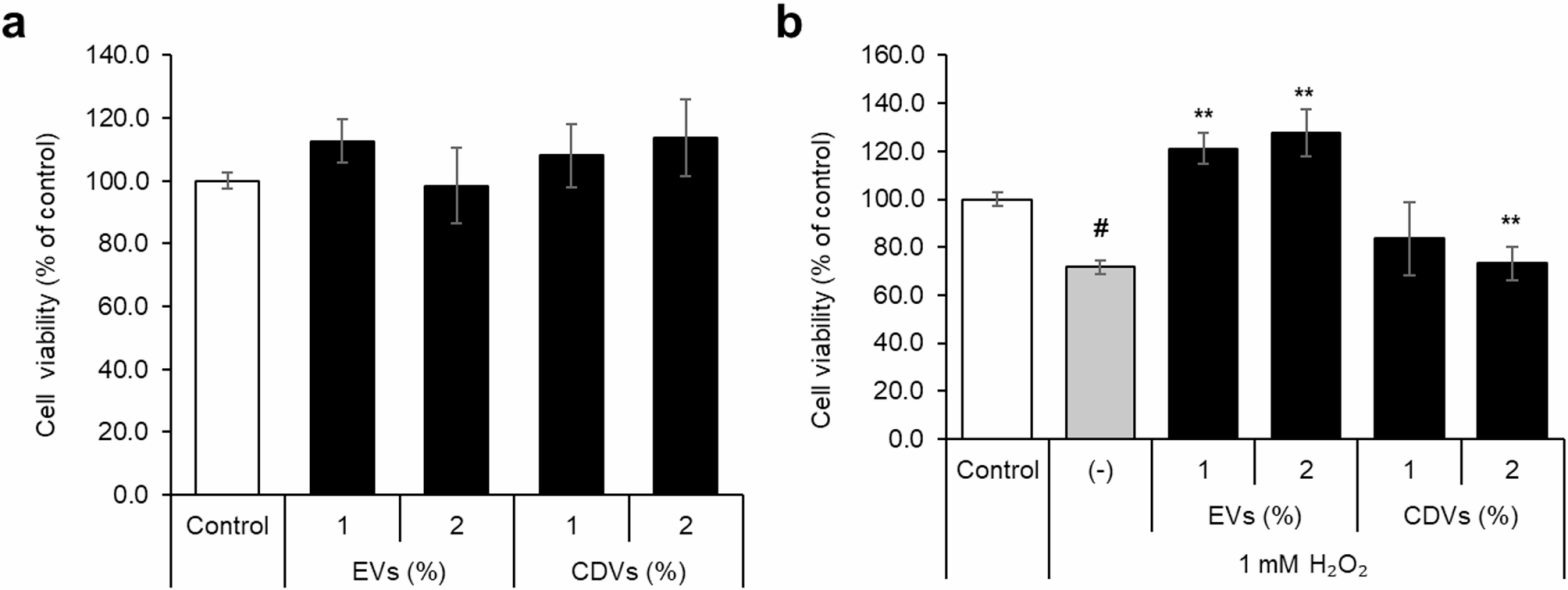
Cytotoxicity and ROS-scavenging capacity of EVs and CDVs in HDF cells. (**a**) Cell viability assessed by MTT assay after treatment with EVs or CDVs. (**b**) ROS-scavenging capacity under oxidative stress induced by 1 mM H₂O₂. Data are presented as mean ± SD of three independent experiments. #*p* < 0.05 vs. untreated control; *p < 0.05, *p* < **0.01 vs. H₂O₂-treated cells.

To assess the protect effect against oxidative stress in cells, human dermal fibroblast (HDF) cells were pre-treated with EVs or CDVs, followed by 1mM H_2_O_2_ exposure. Cytotoxicity was first assessed by MTT assay, confirming no significant cytotoxicity from either vesicle type (Fig. 5a). H_2_O_2_ exposure alone reduced HDF viability to 71.6 ± 2.9% relative to untreated control. Co-treatment with EVs significantly restored cell viability to 121.1 ± 6.5% at 1% and 127.6 ± 9.8% at 2%, while CDVs showed a markedly lower protective effect (Fig. 5b).

**Fig. 6:**
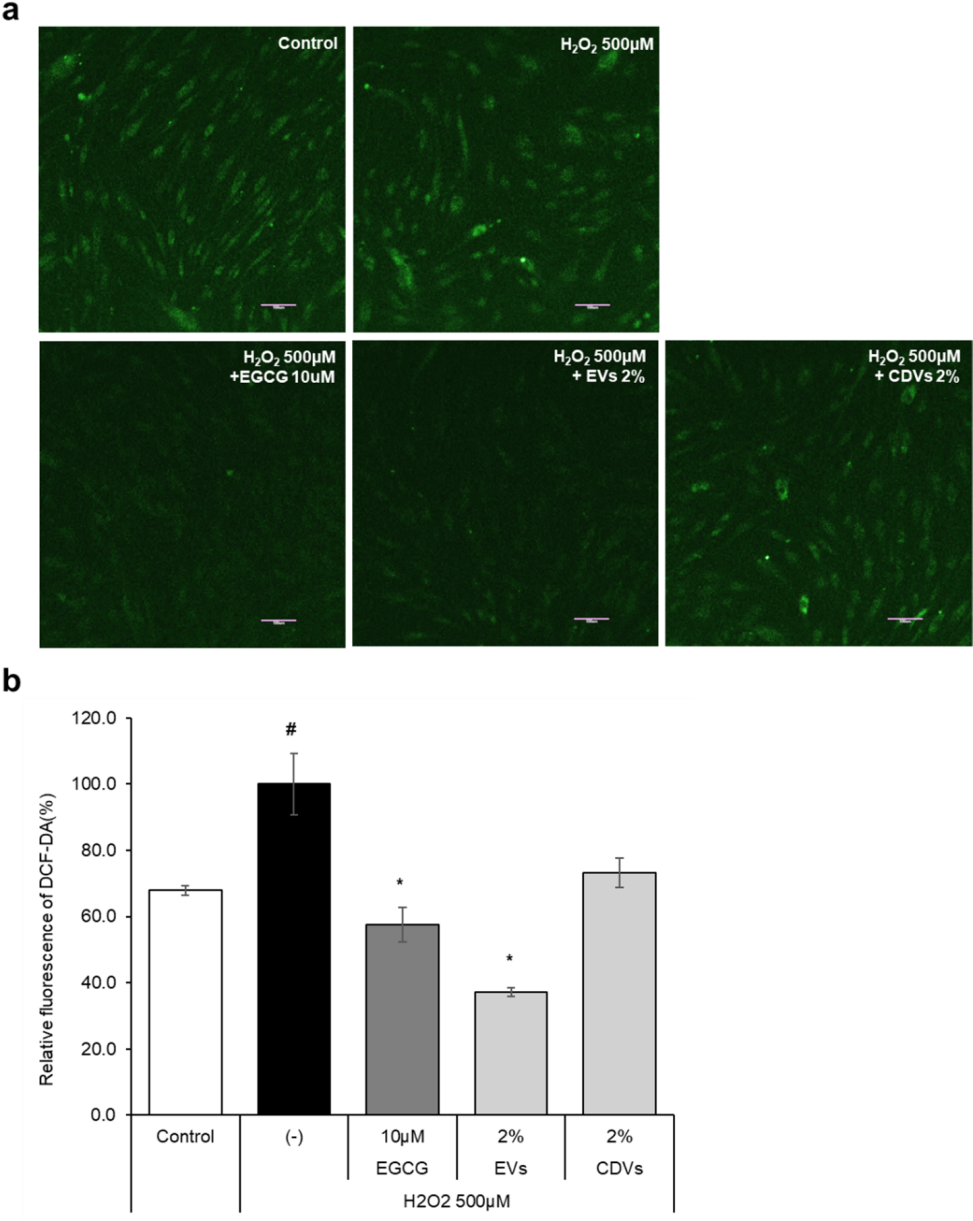
Intracellular ROS-scavenging effect of EVs and CDVs under oxidative stress. (**a**) Representative fluorescence images showing intracellular ROS levels, visualized by H₂DCFDA staining (green fluorescence). (**b**) Quantification of ROS levels based on fluorescence intensity. Data are presented as mean ± SD of three independent experiments. #*p* < 0.05 vs. untreated control; *p < 0.05, **p < 0.01 vs. H₂O₂-treated cells.

Intracellular ROS levels were further assessed using the 2′,7′-Dichlorodihydrofluorescein diacetate (H₂DCFDA) fluorescence assay at a concentration of 2%. EVs significantly reduced H₂O₂-induced ROS production by 62.86 ± 1.3%, which exceed the reduction observed with the positive control EGCG. In contrast, CDVs showed 26.77 ± 4.4% inhibition at the same concentration (Fig. 6a, b). These results were consistent with DPPH assay findings, supporting the superior antioxidant EVs compared to CDVs.

### Effects of EVs and CDVs on LPS-induced NO and PGE₂ production

**Fig. 7:**
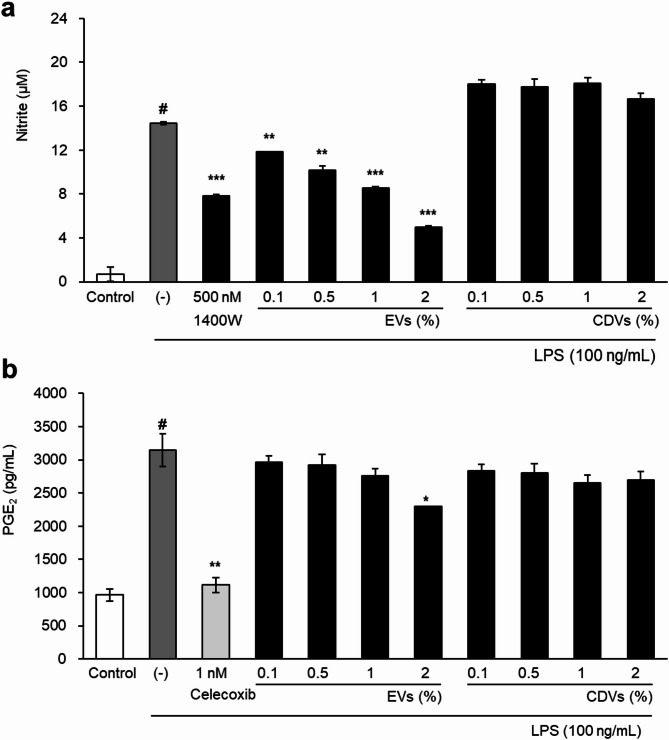
Anti-inflammatory effects of EVs and CDVs in LPS-stimulated RAW 264.7 cells. (**a**) Inhibition of nitric oxide (NO) production. LPS (100 ng/mL) was used to induce inflammation, and 1400 W (500 nM) served as a positive control. (**b**) Inhibition of prostaglandin E₂ (PGE₂) production. Celecoxib (1 nM) was used as a positive control. Data are presented as mean ± SD of three independent experiments. #*p* < 0.05 vs. LPS-untreated control; *p < 0.05, **p < 0.01, ***p < 0.001 vs. LPS-treated control.

The anti-inflammatory activities of EVs and CDVs were assessed by measuring nitric oxide (NO) and prostaglandin E₂ (PGE₂) production in LPS-stimulated RAW264.7 macrophages. Cells were treated with concentration (0.1–2%) of EVs or CDVs following LPS stimulation (100 ng/mL). EVs treatment resulted in a dose-dependent reduction in both NO and PGE₂ levels. At 2%, EVs significantly suppressed NO production to a level comparable to that observed with the selective iNOS inhibitor 1400 W (500 nM) (Fig. 7a). PGE₂ levels were also significantly decreased by EVs treatment in a concentration-dependent manner (Fig. 7b). In contrast, CDVs showed no significant inhibition of NO production and only a modest reduction in PGE₂ levels at the highest concentration tested (Fig. 7a, b). These findings indicate that EVs exhibit more potent anti-inflammatory activity than CDVs under LPS-induced inflammatory conditions.

## Discussion

Both CDVs and EVs are membrane-bound structures enclosed by a lipid bilayer, but differ in their origin, secretion mechanisms, and biological roles. EVs are released through endosomal pathways (e.g., exosomes) or membrane budding (e.g., microvesicles), and are involved in various physiological processes such as immune regulation and tissue repair^14,15^. In contrast, CDVs are generated through artificial disruption methods and primarily reflect the basal cytoplasmic content of cells, without active secretion into extracellular environments.

In this study, we performed proteomic profiling of EVs and CDVs derived from LR to investigate their molecular composition and potential functional differences. The results revealed distinct proteomic signatures between two vesicle types, suggesting functional specialization of EVs.

Notably, HSP70 was detected exclusively in EVs. HSP70 is known as an EVs marker and is primarily associated with the outer membrane surface of exosomes^16–18^. This observation is in agreement with previous reports showing differential expression of common EVs markers such as CD9, CD63, and CD81 between naturally secreted vesicles and artificial vesicles preparations^19^. The distinct surface proteome of EVs, as exemplified by the presence of HSP70, may contribute to their enhanced functional properties by facilitating receptor-mediated interaction, immune modulation, and targeted cellular uptake^20,21^.

Proteomic analysis identified 1,342 proteins across both vesicle types, with 1,228 proteins shared and 114 proteins unique to CDVs, indicating 91.5% overlap. This substantial overlap suggests a common cellular origin. In contrast, the presence of CDV-specific proteins may reflect differences in vesicle biogenesis, particularly in cargo incorporation. CDVs generated by high pressure-induced membrane disruption are likely to incorporate cellular components non-selectively, rather than through active, machinery-driven sorting processes. Unlike EVs, this method bypasses the regulated secretion pathways that are essential for exosome biogenesis. Principal component analysis further distinguished the two vesicle types, with PC1 accounting for 70.33% of the variance, confirming their molecular distinctiveness. A total of 151 proteins were differentially expressed between EVs and CDVs, with 70 proteins upregulated and 81 downregulated in EVs. Several proteins enriched in EVs were involved in signal transduction, environmental sensing, and transmembrane transport. Notably, *arlS* (log₂FC = 6.11) and *hisF* (log₂FC = 6.29), associated with bacterial two-component systems and histidine biosynthesis, were highly expressed in EVs, suggesting potential roles in adaptive signaling and microbial niche modulation. In contrast, proteins downregulated in EVs were primarily linked to ribosomal function, translation, and DNA repair, indicating a relative depletion of housekeeping functions. Similar protein distribution patterns have been reported in previous studies comparing naturally secreted EVs and artificially derived vesicles^19,22,23^. Taken together, these results suggest that EVs possess a more specialized proteomic profile tailored for intercellular signaling and environmental adaptation, whereas CDVs reflect a broader, less regulated sampling of cytoplasmic contents.

The identified differential protein profiles between EVs and CDVs highlight distinct vesicle biogenesis and functional properties. Although HSP70 was present in our proteomic dataset in both EVs and CDVs, it was not identified as differentially expressed. Western blot analysis, however, revealed detectable levels of HSP70 predominantly in EVs. This difference could be attributed to structural or compositional factors influencing accessibility or surface localization of HSP70 on EVs. Further studies are required to clarify these possibilities, and thus this interpretation is not included in the current manuscript to avoid undue speculation. Furthermore, the enriched expression of signaling-related proteins in EVs suggests their potential superior antioxidative and anti-inflammatory activities.

GO enrichment analysis supported these observations, showing significant enrichment of proteins involved in the two-component regulatory system and stress response in EVs. These functional categories are associated with microbial environmental adaptation and intercellular communication. Such enrichment may be associated with the superior antioxidant and anti-inflammatory effects observed in EVs compared to CDVs. Additionally, the presence of ribosomal proteins, ribonucleoproteins, ligases, and repressors in EVs indicates regulatory potential beyond simple protein transport. This observation aligns with growing evidence that EVs serve as vesicles for functional biomolecules, actively participating in intercellular signaling networks through the delivery of regulatory proteins to recipient cells^24^. The expression patterns also highlight the functional specialization of EVs for signaling and adaptation. In addition to the substantial overlap in identified proteins between EVs and CDVs, our analysis revealed that 114 proteins were uniquely present in CDVs. GO enrichment demonstrated that these unique proteins are largely associated with ribosomal structure, translation, and nucleic acid metabolism reflecting core cellular and housekeeping functions (ex. rplQ, xseB). This compositional variation is likely a consequence of the non-selective cargo incorporation during CDVs generation through mechanical disruption, in contrast to the active and selective sorting mechanisms that drive EVs biogenesis. The functional profile of these CDVs unique proteins suggests that their presence is less likely to confer specialized bioactivity, particularly in the context of cell signaling, stress adaptation, or membrane-mediated interactions that are enriched in EVs. Consequently, while CDVs mirror the cytoplasmic composition of their source cells, they lack the specific enrichment of proteins implicated in antioxidants and anti-inflammatory signaling, which may explain their relatively limited activity in our in vitro assays.

Oxidative stress is a pathological condition resulting from an imbalance between ROS production and antioxidant defense systems^25^. It can be triggered by excessive ROS generation or insufficient clearance mechanisms. The skin, as the primary barrier exposed to the external environment, is particularly susceptible to ROS induced damage, which contributes to inflammation, photoaging (premature skin aging), immunosuppression, carcinogenesis, and apoptosis^26^. In this study, EVs exhibited superior antioxidant and anti-inflammatory effects compared to CDVs in multiple in vitro assays. These functional differences are in line with the distinct proteomic profiles of EVs, which were enriched in proteins related to stress response and environmental adaptation. This distinct proteomic profile suggests that EVs are selectively loaded with functional proteins that facilitate enhanced host-microbe interactions and stress responses. NADP-dependent oxidoreductase (A0A508Z1V3) and MenA play crucial roles in microbial survival and metabolic processes, and their functions may be indirectly linked to the regulation of oxidative stress and host-microbe interactions. Specifically, NADP-dependent oxidoreductase catalyzes the consumption or production of reduced nicotinamide adenine dinucleotide phosphate (NADPH), a vital cofactor for glutathione reductase, an essential antioxidant enzyme system responsible for detoxifying reactive oxygen species (ROS) within cells^27,28^. Additionally, MenA is a key enzyme in the biosynthesis of vitamin K2 (menaquinone), derivatives of which have been reported to possess anti-inflammatory properties, potentially benefiting host cells^29^. Similar findings have been reported in EVs derived from other probiotic strains, where specific protein cargoes were linked to antioxidant effects^30^. The anti-inflammatory activity of EVs may be associated with their unique protein cargo involved in immune regulation. Previous studies have shown that *Lactiplantibacillus plantarum*-derived EVs suppress inflammatory responses and promote M2 macrophage polarization^31^, which is consistent with the functional effects observed in our study. In addition, emerging evidence suggests that cells exposed to external stressors regulate adaptive responses through dynamic modulation of exosome biogenesis and cargo composition, thereby enhancing oxidative stress resistance and attenuating apoptotic pathways. This stress-induced exosomal reprogramming is considered a key mechanism for maintaining cellular homeostasis under adverse conditions^32,33^. The enrichment of stress response-related proteins in EVs observed in our proteomic analysis may contribute to their enhanced antioxidant capacity. Emerging evidence highlights the role of exosomal signaling in immune modulation, particularly through anti-inflammatory mechanisms. Exosomes have been shown to mediate intercellular communication by delivering bioactive molecules that regulate immune responses^34,35^. In our study, EVs were enriched in proteins associated with signal transduction which may contribute to their immunomodulatory properties by interacting with immune cell receptors and influencing downstream signaling pathways. The superior functional activity of naturally secreted EVs, compared to artificially generated CDVs, has potential implications for therapeutic development. Our data suggests that the natural biogenesis of EVs may allow for more selective and functional packaging of immune- and stress-related molecules, which could be compromised in artificial vesicle production. While this study offers meaningful insights into the proteomic distinctions between EVs and CDVs, further investigation is warranted to fully elucidate their functional mechanisms, particularly in the context of skin-related applications. Additional studies focusing on the role of non-protein components (e.g. lipids and nucleic acids) and the interactions between vesicle cargo and host cells will be essential to advance our understanding and therapeutic utilization of probiotic-derived vesicles.

In conclusion, this study provides a comprehensive proteomic comparison of EVs and CDVs from LR, revealing distinct molecular signatures between two vesicle types. EVs were enriched in proteins associated with signal transduction, environmental adaptation, and stress response, which may contribute to their enhanced antioxidant and anti-inflammatory properties. These findings improve our understanding of the molecular characteristics of probiotic-derived vesicles and offer insights that may support their application in therapeutic and cosmetic contexts. The observed functional advantages of naturally secreted EVs highlight the importance of biogenesis pathways in determining vesicle functionality. Furthermore, these findings may also be applied to the development of EVs engineered to exhibit specific functional properties.

## Materials & methods

### Bacterial culture and growth conditions

LR BS-Pro-8 (accession number: KCTC 15876BP), obtained from BioSpectrum Fermentation Institute (Osan-si, South Korea), was used in this study. The strains were isolated from water kefir. Water kefir samples were immersed in sterile distilled water and incubated statically at 37 °C for 1 h. The resulting soaking solution was serially diluted in sterile physiological saline, and each dilution was spread onto MRS agar plates (Difco). The plates were incubated at 37 °C for 48 h. Colonies exhibiting morphological characteristics typical of lactic acid bacteria were streaked onto MRS agar supplemented with 2% (w/v) calcium carbonate and further incubated. Colonies producing clear zones were subcultured on fresh MRS agar plates through successive passages to obtain purified single isolates. For taxonomic identification, 16 S rRNA gene sequencing was performed on the purified isolates (KCTC 15876BP). After fermentation, the culture was centrifuged at 5,000 rpm for 30 min at 4 °C to separate the supernatant from the bacterial cells.

### Isolation of EVs and CDVs

To isolate EVs, the cell-free supernatant was first concentrated by tangential flow ultrafiltration using a 100 kDa cutoff polyethersulfone (PES) membrane (Merck, Darmstadt, Germany). The concentrate was then filtered through a 0.22 μm PES membrane filter (Jet Bio-Filtration Co., Ltd., Guangzhou, China). The purified EVs were stored at − 80 °C until further use.

For the isolation of CDVs, bacterial cells were resuspended in triple-distilled water (3DW) and subjected to high-pressure homogenization (Scientz-150, Ningbo Scientz Biotechnology Co., Ltd., Ningbo, China) at 1,000 bar for three cycles. The resulting suspension was centrifuged at 5,000 rpm for 30 min at 4 °C to remove cellular debris. The supernatant was then concentrated using a 100 kDa molecular weight cut-off (MWCO) PES membrane, following the same protocol used for EVs. The concentrate was subsequently filtered through a 0.22 μm PES membrane. The final CDVs preparation was stored at − 80 °C for subsequent analysis.

### Nanoparticle characterization by nanoparticle tracking analysis (NTA) and transmission electron microscopy (TEM)

To assess the size distribution and particle concentration of EVs and CDVs, NTA was performed using a ZetaView (PMX-120, ParticleMetrix GmbH, Inning am Ammersee, Germany). Prior to measurement, the instrument’s camera and laser were aligned, and automatic symmetry correction was applied. Samples were diluted to achieve an optimal concentration of 100–200 particles per frame. Measurements were conducted at 11 different positions with the following settings: camera sensitivity 80, shutter speed 100, video quality set to medium at 30 frames per second, minimum particle area of 10, and maximum particle area of 1,000. Measurements were performed at 23.5 °C, and data were analyzed using ZetaView software.

For morphological assessment, EVs and CDVs samples were applied to 300-mesh copper grids and negatively stained with 2% (w/v) uranyl acetate. Imaging was performed using a TEM (JEM-2100Plus, JEOL Ltd., Tokyo, Japan) operated at an accelerating voltage of 200 kV.

### Western blot analysis

Thirty micrograms of protein extracted from EVs and CDVs were loaded on a 4–12% gradient SDS-PAGE gel (NuPAGE™, Invitrogen, Carlsbad, CA) and then transferred onto PVDF membranes (iBlot™, Invitrogen). Membranes were blocked with a 5% (w/v) skim-milk (BD Difco, Sparks, MD) prepared in 1X TBS-T solution (10X TBS with Tween 20, Biosesang, Yongin-si, South Korea). Rabbit polyclonal Antibody against HSP 70 (Agrisera, Sweden) were diluted 1:1000 in 5% (w/v) skim-milk in 1X TBS-T and incubated with the membranes overnight at 4 °C. After washing, the blots were incubated with HRP-conjugated goat anti-rabbit secondary antibody (Cell signaling, Frankfurt, Germany). Signals were detected using ECL substrate (SuperSignal™ West Pico PLUS Chemiluminescent Substrate, Thermo Scientific, Rockford, IL, USA) according to the manufacturer’s instructions. Chemiluminescent images were acquired using ImageQuant LAS 500 sysmtem (GE Healthcare Biosciences, Uppsala, Sweden).

### Proteomics analysis

#### Protein extraction

Samples were resuspended in 8 M urea/100 mM ammonium bicarbonate to a final concentration of 10 mg/mL. The resuspended samples were then sonicated for 10 min at 10 °C and centrifuged at 16,000 × g for 5 min at 4 °C. The resulting supernatants were carefully transferred to fresh tubes for further processing.

#### Reduction, alkylation, and digestion

Proteins were reduced with 10 mM dithiothreitol (DTT) at 37 °C for 30 min and alkylated with 25 mM iodoacetamide at room temperature for 30 min in the dark. The urea concentration was then diluted to below 1 M using 100 mM ammonium bicarbonate. Proteins were digested with sequencing-grade trypsin (enzyme-to-protein ratio of 1:25; Promega, San Luis Obispo, CA) at 37 °C for 16 h. The digestion reaction was quenched by adding 1% trifluoroacetic acid (TFA), and the resulting peptides were desalted using SOLA HRP C18 cartridges (30 mg/2 mL; Thermo Scientific). Desalted peptides were vacuum-dried using a SpeedVac concentrator and reconstituted in 100 mM triethylammonium bicarbonate. Tandem Mass Tag (TMT) 6-plex reagents (Thermo Scientific) were equilibrated at room temperature for 5 min, and any residual moisture was removed from the tube walls before dissolving each tag in 41 µL of acetonitrile. The dissolved TMT reagents were added to the individual peptide samples and incubated at room temperature for 1 h. Labeling reactions were terminated by adding 8 µL of 5% hydroxylamine to each sample. Labeled peptides from all six samples were pooled into a single tube, vacuum-dried, and reconstituted in 0.1% TFA. To remove unbound TMT reagents, the pooled mixture was desalted using SOLA HRP 96-well plate C18 cartridges (30 mg/2 mL; Thermo Scientific). The final desalted peptides were vacuum-dried again and reconstituted in 0.1% TFA for subsequent LC-MS/MS analysis.

#### LC-MS/MS analysis

LC-MS/MS analysis was performed using a Thermo Dionex Ultimate 3000 nano-LC system coupled to a Thermo Orbitrap Exploris 480 mass spectrometer. Peptides were separated on a PepMap™ RSLC C18 analytical column (75 μm inner diameter × 50 cm length) maintained at 50 °C, using a 180 min gradient ranging from 2% to 95% acetonitrile containing 0.1% formic acid and 5% DMSO. Raw data were analyzed using SAGE software (version 0.14.7) against the LR UniProt database (ID: 47715). Proteins with *p* < 0.05 and fold changes above the 95th percentile (upregulated) or below the 5th percentile (downregulated) were defined as DEPs.

#### Gene ontology enrichment analysis

Gene Ontology (GO) enrichment analysis was performed using DAVID database. Enriched GO terms in the categories of biological process, molecular function, and cellular component categories were identified using a significance threshold of *p* < 0.1.

### DPPH radical scavenging assay

The antioxidant activity of the sample was evaluated using the DPPH (1,1-diphenyl-2-picrylhydrazil, Sigma, St. Louis, MO) radical scavenging assay. The test sample was dissolved in methanol. A volume of 130 µL of each test solution was mixed with 130 µL of 200 µM DPPH solution. The mixtures were incubated at room temperature in the dark for 30 min. After incubation, the remaining DPPH was quantified by measuring the absorbance at 517 nm using a microplate reader (Powerwave X, Bio-Tek Instruments Inc., Winooski, VT). The antioxidant capacity of the sample was quantified as a percentage reduction in absorbance relative to the control group, indicating the extent of radical scavenging activity.

$${\text{Radical Scavenging Activity}}\left( \% \right){\text{ }}={\text{ }}\left( {{\text{1 }}-{\text{ Absorbance of test material }}/{\text{Absorbance of control}}} \right){\text{ }} \times {\text{ 1}}00$$

### Intracellular reactive oxygen species (ROS) assay in human dermal fibroblast (HDF) cells

Primary Dermal Fibroblast Normal; Human, Neonatal (HDF, PCS-201-010™) were obtained from ATCC (American Type Culture Collection, Manassas, VA) and cultured in Dulbecco’s Modified Eagle’s Medium (DMEM, Welgene, Daegu-si, South Korea) with 10% fetal bovine serum (Welgene) and penicillin/streptomycin (100 IU/50 µg/mL) at 37 °C with 5% CO₂. Cell viability was assessed using MTT assay. HDF cells were seeded at a density of 1 × 10⁵ cells per well in a 96-well plate, followed by a 24 h incubation for cell adherence. Subsequently, cells were treated with H₂O₂ in the presence or absence of EVs or CDVs and further incubated for 24 h. After treatment, cells were incubated with MTT reagent (0.1 mg/mL in DMEM) for 2 h. The MTT solution was then carefully removed by aspiration, and the resulting formazan crystals were solubilized by adding 200 µL of DMSO to each well. Absorbance was measured at 570 nm using a microplate reader.

The intracellular ROS levels were assessed using the H_2_DCFDA fluorescent dye (Invitrogen). HDF were seeded in 6-well cell culture plate at a density of 1 × 10^6^ cells per well and incubated for 24 h. Subsequently, cells were treated with test substances in serum-free medium overnight. After treatment, the medium was replaced with serum-free medium containing 30 µM H_2_DCFDA and cells were incubated for an additional 1 h. The cells were then washed twice with PBS, followed by treatment with 500 µM H₂O₂ for 30 min. The generation of intracellular ROS was visualized by capturing fluorescence images using a fluorescence microscope (Nanoscope systems, Daejeon-si, South Korea). For quantitative fluorescence measurement, cells were lysed with 1 N NaOH, and fluorescence intensity (Excitation at 485 nm, Emission at 535 nm) was measured using a microplate reader (Tecan, Oberdiessbach, Switzerland).

#### Measurement of NO and PGE₂ in RAW 264.7 cells

The murine macrophage cell line RAW264.7 (Korean Cell Line Bank, Seoul-si, South Korea) was cultured in DMEM (Welgene) at 37 °C with 5% CO₂. To evaluate the anti-inflammatory effects, cells were pretreated with EVs and CDVs at concentrations of 0.1%, 0.5%, 1%, and 2% for 1 h, followed by stimulation with lipopolysaccharide (LPS, Sigma, St. Louis, MO). LPS was then added at a final concentration of 100 ng/mL and incubated for 24 h. After incubation, the culture supernatants were collected to measure the level of the inflammatory mediator nitric oxide (NO). Griess reagent was prepared by mixing equal volumes of 1% (w/v) sulfanilamide (Sigma) dissolved in 5% phosphoric acid (DUKSAN, Ansan-si, South Korea) and 0.1% (w/v) N-(1-Naphthyl)ethylenediamine dihydrochloride (Sigma) dissolved in distilled water. The culture supernatants were reacted with an equal volume of Griess reagent, and the amount of nitrite, a stable NO metabolite, was quantified by measuring the absorbance at 540 nm.

To determine the level of Prostaglandin E_2_ (PGE_2_) in the same supernatants, the Prostaglandin E_2_ Parameter Assay Kit (R&D Systems, Minneapolis, MN) was used. Absorbance was measured at 450 nm.

### Statistical analysis

In this experiment, the experiment was repeated three times, and the results were expressed as the average value. Statistical significance was verified at a confidence level of *p* < 0.05 using student’s t-test (*p < 0.05, **p < 0.01, ***p < 0.001).

## Supplementary Information

Below is the link to the electronic supplementary material.

Supplementary Material 1

Supplementary Material 2

Supplementary Material 3

## Data Availability

The proteomic raw data used in this study are available via ProteomeXchange with identifier PXD065977.
