# Supplementary material for: Functional and proteomic analysis of Lactobacillus rhamnosus-derived extracellular vesicles with antioxidant and anti-inflammatory activity
Source: Sci Rep. 2025 Dec 18;16:3124. doi: 10.1038/s41598-025-32989-6 (PMC12830880; doi:10.1038/s41598-025-32989-6)
Supplement: Supplementary file 1 — Supplementary Material 1 [file 41598_2025_32989_MOESM1_ESM.docx]

**Supplementary Figure and Table Legends**

**Supplemental Figure S1.** Full membrane images showing two independent preparations of both vesicle type (EVs and CDVs) loaded onto the same gel. Lanes labeled Set 1 and Set 2 represent separate isolations of EVs and CDVs. The main figure (Figure 1d) in the manuscript displays cropped regions from Set 2.

**Supplemental Table S1.** Table lists the median expression levels (log2) of proteins that were identified as differentially expressed between EVs and CDVs.
