# Supplementary figures and images for: Functional and proteomic analysis of Lactobacillus rhamnosus-derived extracellular vesicles with antioxidant and anti-inflammatory activity
Source: Sci Rep. 2025 Dec 18;16:3124. doi: 10.1038/s41598-025-32989-6 (PMC12830880; doi:10.1038/s41598-025-32989-6)

**Supplemental Figure S1.**  
**(Full Membrane Images for Cropped Data in Figure 1d)**

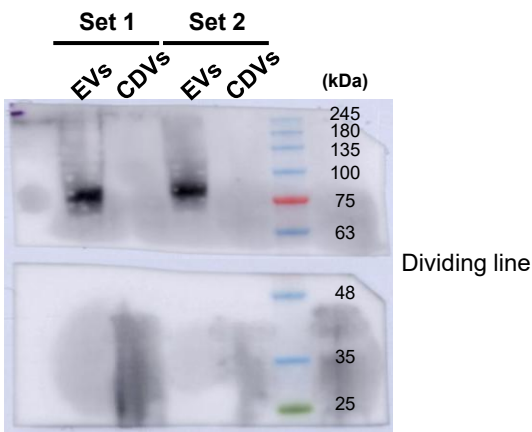

Supplement: Supplementary file 2 — Supplementary Material 2 [file 41598_2025_32989_MOESM2_ESM.pdf]
