# Supplementary material for: Functional and proteomic analysis of Lactobacillus rhamnosus-derived extracellular vesicles with antioxidant and anti-inflammatory activity
Source: Sci Rep. 2025 Dec 18;16:3124. doi: 10.1038/s41598-025-32989-6 (PMC12830880; doi:10.1038/s41598-025-32989-6)
Supplement: Supplementary file 3 — Supplementary Material 3 [file 41598_2025_32989_MOESM3_ESM.pdf]

Supplemental Table S1.
(Median expression levels (log2) of differentially expressed proteins in extracellular vesicles (EVs) and cell-derived vesicles (CDVs).)

No.	Protein ID	Gene	Description	EVs	CDVs
**Up-regulated proteins**					
1	A0A171J904	hisF	Kinase, L-histidine biosynthetic process	19.5492628	13.2544769
2	A0A6N3BJ42	arlS	Kinase, Transferase, key signal transducers	18.9557137	12.844417
3	A0A508YJH6	nrnA	Nucleic acid binding, Microbial metabolism in diverse environments	17.0728597	12.9721718
4	A0A508Z1V3		NADP-dependent oxidoreductase	14.9473496	11.2405925
5	A0A249DBX2		MFS Transporter, Transmembrane transporter activity	16.1895934	12.6111747
6	A0A0M2G5F9		DUF2140 domain-containing protein	16.0157012	12.569299
7	A0A508Z0V2	pepV	Dipeptidase activity	17.1855681	13.7715629
8	A0A0D6U9N8		Sugar transport	16.8281349	13.5242697
9	A0A7X2J725	lysM	Binds to peptidoglycan, Cell wall binding	16.0621025	12.7804506
10	A0A508YSK0	menA	1,4-dihydroxy-2-naphthoate polyprenyltransferase	15.8542767	12.6547385
**Down-regulated proteins**					
1	A0A7S7JFA0		Membrane protein	14.0682454	17.7278342
2	A0A0D6U8P4	rplP	rRNA binding, tRNA binding, Translation	12.9455604	16.2748091
3	A0A0D6U9Y8	rplQ	Structural constituent of ribosome, Tranlation	14.0561174	17.2836822
4	A0A809N128	rplW	rRNA binding, Structural constituent of ribosome, Translation	13.2786108	16.4943879
5	A0A2A5L762	xseB	Exodeoxyribonuclease	16.1471846	19.2791118
6	A0A2A5L2X5		RNA binding	13.7976748	16.9239729
7	A0A0D6U5L6	nusG	Transcription	13.9683345	16.8695414
8	A0A2A5L5B0		DNA binding, Transcription regulator	12.6344887	15.4448191
9	A0A180C927	lepB	Signal peptide processing	13.0765806	15.8715261
10	A0A171J940	msrA	Protein modification process, Oxidoreductase	13.8612556	16.6131326
